# A Case of Giant Hepatic Hydatid Cyst Infected with *Morganella morganii* and the Literature Review

**DOI:** 10.1155/2012/591561

**Published:** 2012-11-11

**Authors:** Ismail Necati Hakyemez, Mustafa Sit, Gulali Aktas, Tekin Tas, Fırat Zafer Mengeloglu, Abdulkadir Kucukbayrak

**Affiliations:** ^1^Department of Infectious Diseases and Clinical Microbiology, Faculty of Medicine, Abant Izzet Baysal University, 14280 Bolu, Turkey; ^2^Department of General Surgery, Faculty of Medicine, Abant Izzet Baysal University, 14280 Bolu, Turkey; ^3^Department of Internal Medicine, Faculty of Medicine, Abant Izzet Baysal University, 14280 Bolu, Turkey; ^4^Department of Medical Microbiology, Faculty of Medicine, Abant Izzet Baysal University, 14280 Bolu, Turkey

## Abstract

Hydatid cyst disease is a common worldwide zoonosis. Most of the cysts are located in the liver. Abscess formation due to infection of the cyst is an important complication. *M. morganii,* a Gram-negative *Bacillus*, is a quite rare cause of liver abscess. A 77-year-old woman was admitted to hospital with complaints of fever, chills, nausea, vomiting, loss of appetite, and abdominal pain located in the right-upper quadrant. Her history was positive for hepatic hydatid cyst disease ten years ago. Physical examination revealed a painful mass filling the right-upper quadrant and extending down to umbilicus. Indirect hemagglutinin test for hydatid cyst was positive at a titer of 1/320. Giant liver abscess due to infected hydatid cyst was found in computed tomography scan. Surgeons performed cystectomy and cholecystectomy. Cefazoline, cefuroxime, and metronidazole were administered empirically, but all the three agents were replaced with intravenous ceftriaxone after *M. morganii* was isolated from the cultures of the abscess material. Clinical signs of the patient resolved at the second week of treatment, and she was discharged.

## 1. Introduction

Turkey is among the countries where echinococcosis is endemic [1]. Patients with hepatic hydatid cyst remain asymptomatic for a long time in the course of the disease. Patients are admitted to hospital usually with clinical signs related to increased size or the complications of the cyst. Infection of the cyst causes pyogenic liver abscess (PLA). Secondary acute peritonitis may occur as a complication in some cases if the abscess ruptures into the abdominal cavity [2]. *M. morganii* is a Gram-negative *Bacillus* commonly found in the environment and in the normal intestinal flora in human. It may cause systemic infections [3]. In this paper, we present a PLA case caused by the infection of hepatic hydatid cysts with *M. morganii*, which is a rare infectious agent. 

## 2. Case Report

**Figure 1:**
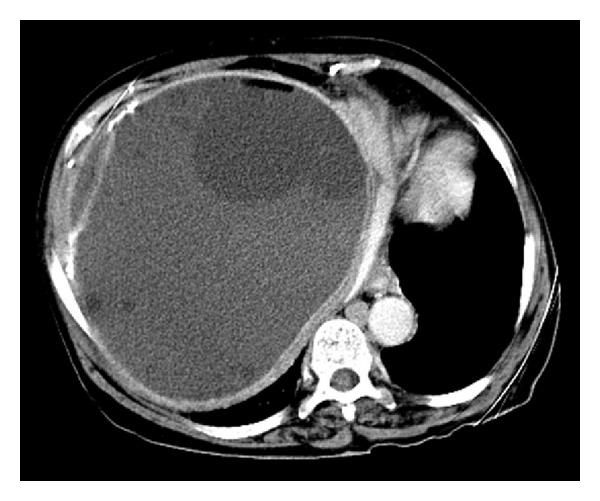
CT image of the thick-walled, complicated multicystic lesion and air density in it with a dimension of 19 cm × 19 cm × 20 cm.

A 77-year-old woman was admitted to the Emergency Department of Abant Izzet Baysal University Hospital with complaints of fever, chills, nausea, vomiting, loss of appetite and abdominal pain located in the right-upper quadrant. She described fever and chills, especially in the night time for the last two weeks before admission. Her history was positive for hepatic hydatid cyst ten years ago. Physical examination revealed a mild, confused, and sick appeared woman. Vital signs were as follows: temperature 39.2°C, pulse rate 122 beats/min, respiration rate 20 breaths/min, and blood pressure 95/50 mmHg. Abdominal examination revealed a painful mass filling the right-upper quadrant and extending down to umbilicus. Laboratory findings were as follows: total white cell count 7.6 × 10^9^/L (90% neutrophil), hemoglobin 10.9 g/dL, platelet 121000/mm^3^, blood urea 77 mg/dL, creatinine 0.7 mg/dL, aspartate aminotransferase 47 U/L, alanine aminotransferase 19 U/L, alkaline phosphatase 76 U/L, and C-reactive protein (CRP) 265 mg/dL. Indirect hemagglutination (IHA; Fumouze Diagnostics, France) test for hydatid cyst was positive at a titer of 1/320. Ultrasonography (US) of the abdomen revealed a hyperechogenic lesion filling the right-upper quadrant with a dimension of 19 cm × 19 cm × 20 cm. The lesion included more than 10 cysts in it, and the largest cyst was with an 80 mm diameter. Complicated, thick-walled multicystic lesions with different dimensions and with air-fluid levels located in right-upper quadrant of the abdomen were determined in abdominal computed tomography (CT) (Figure 1). The patient was diagnosed with PLA secondary to the infection of hepatic hydatid cysts. Surgeons performed cystectomy and cholecystectomy. Abscess material was cultured into microbial broth. Intravenous cefazolin 3 × 1 g and 3 × 500 mg metronidazole were empirically administered, and cefazolin was replaced with 2 × 750 mg intravenous cefuroxime because no clinical improvements were achieved. Gram staining of the abscess material revealed 5-6 leukocytes and Gram-negative *bacilli*. Gram-negative growth in the culture was identified as *M. morganii* by conventional methods and by VITEK 2 automated system (bioMerieux Inc., Marcy L'etoil, France). Antibiotic susceptibility tests were done with VITEK 2 (bioMerieux Inc., Marcy L'etoil, France) automated system. The *bacilli* were susceptible to ceftriaxone, ceftazidime, ertapenem, imipenem, meropenem, tetracycline, aztreonam and trimetoprim/sulphametoksazol. The patient was consulted to infectious diseases specialists in the 7th day and according to their advice, antibiotherapy was switched to ceftriaxone 2 × 1 gram daily. Clinical signs of the patient resolved at the second week of treatment and she was discharged from hospital. 

## 3. Discussion

Hydatid cyst disease, a common worldwide zoonosis, is an important public health issue in our country. Approximately 65% of the cysts are located in the liver. Abscess formation due to infection of the cyst is an important complication of the disease [3]. Although it is rare, PLA can be fatal. Global incidence is about 1.1–2.3 per 100.000 cases [4, 5]. PLA is more common in men aged 50–60 years. Most of the cases are cryptogenic, and some of them are associated with biliary tract diseases at present time [6]. Epidemiologic risk factors are as follows: diabetes mellitus (leading risk factor), alcoholism, immune deficiency, malignancy, and liver transplantation [7, 8]. The patient we present had hepatic cyst hydatid for 10 years. 

Microbial etiology of the PLA is usually polymicrobial (20–50%). The most common microorganisms are *Escherichia coli*, *Klebsiella pneumoniae*, *Proteus* spp., *Pseudomonas* spp., *Streptococci,* and *Enterococci* [8, 9]. Cases with anaerobic microorganisms such as *Bacteroides fragilis* and *Fusobacterium necrophorum* are increasing in number due to developments in diagnosis and culture methods. We determined *M. morganii* in the culture of abscess material in our case. We did not determine any anaerobic agents because we could not culture anaerobically. However, the possibility of having an anaerobic agent is less likely because our case did not respond to metronidazole treatment. The rate of positive blood culture in patients with PLA is approximately 50% [6]. We could not assess the possible bacteremia because we have not made blood cultures during hospitalization.

*M. morganii* is a very rare cause in cases with PLA. It is a Gram-negative *Bacillus* of the environmental and the normal intestinal flora in human and a member of Enterobacteriaceae family [10]. Polymicrobial infection depends on the site of infection. *M. morganii* was first described in the late 1930s as a pathogen in urinary infections [3]. Liver has two blood streams, one from the guts via portal vein and the other from sterile arterial blood stream. Temporary bacteremia in the portal vein is not unusual. Therefore, gut is the main source of liver abscess caused by *M. morganii* [11]. Although it is a common environmental bacteria, it rarely causes community-acquired infections. It causes nosocomial infections usually after surgery [12]. It may cause particularly urinary infections, bacteremia, skin and soft tissue infections, meningitis, ecthyma gangrenosum, spontaneous bacterial peritonitis, chorioamnionitis, septic arthritis, and endophthalmitis [3]. 

*M. morganii* has intrinsic resistance to oxacillin, ampicillin, amoxicillin, most of the first- and second-generation cephalosporins, macrolides, lincosamides, glycopeptides, fosfomycin, fusidic acid, and colistin. However, it is naturally sensitive to aztreonam, aminoglycosides, antipseudomonal penicillins, third- and fourth-generation cephalosporins, carbapenems, quinolones, trimethoprim-sulfamethoxazole, and chloramphenicol [13]. *M. morganii* may develop resistance to multiple antibiotics by a variety of mechanisms, such as the production of inducible extended-spectrum beta-lactamase [14]. In our case, bacteria were sensitive to ceftriaxone, ceftazidime, ertapenem, imipenem, meropenem, tetracycline, aztreonam, and trimethoprim/sulfamethoxazole. 

The most frequent signs and symptoms in PLA are fever, nausea, and abdominal pain located in the right-upper quadrant. Hepatomegaly may accompany in 25% of cases. In most cases, leukocytosis, left shift, elevated transaminase levels, and CRP are common laboratory findings [8, 15]. In our case, patient described fever and chills, nausea, vomiting, loss of appetite, and abdominal pain located in the right-upper quadrant, and physical examination revealed a painful mass filling the right-upper quadrant and extending down to umbilicus. Although leukocyte count was normal, there was a neutrophil domination in hemogram. Liver enzymes were normal, but CRP was extremely elevated. 

US is the cheapest and most commonly used imaging technique in patients suspected with PLA. Contrast-enhanced abdominal CT is more sensitive than US and the gold standard method for the diagnosis of PLA [16]. In our case, the patient was diagnosed with PLA with clinical findings accompanied by the appearance of a giant liver abscess which was consistent with hydatid cyst infection in abdominal US and contrast-enhanced CT.

PLA usually has a good prognosis [3]. The most common complications include pneumonia, sepsis, and septic shock [17]. Mortality rate depends on the age of the patient and comorbidities. It has different outcomes in young and older populations. A study in the literature pointed that clinical outcomes are better, but length of stay in hospital is longer in older population compared to younger population [18]. Its mortality rate has been reduced as a consequence of the improvements in imaging techniques and in the quality of critical care and the use of antibiotics with more extended spectrum. Mortality rate reported from developed countries is about 2–12% [4, 19]. Our case was discharged from hospital after full recovery. 

The effective treatment of the PLA includes appropriate antimicrobial therapy and US-, and CT-guided percutaneous or surgical drainage of abscess. The clinical success rate of antibiotic therapy alone is low. Antibiotic therapy and abscess drainage are necessary in abscesses larger than 5 cm, but antibiotics alone may be sufficient in patients with smaller abscesses [6]. Empirical therapy should be chosen according to the underlying disease and the possible pathogenic microorganism [20]. Empiric antibiotic with broad spectrum should be administered until culture results are obtained. Combination of third-generation cephalosporins and metronidazole should be the first choice for possible polymicrobial anaerobic causes. Treatment should be reassessed according to the culture results. Antimicrobial therapy should be given intravenously in the first two weeks and orally for the following six weeks [6]. The combination of cefazolin and metronidazole was started empirically in our case. Then cefazoline was switched to cefuroxime. The patient was consulted to infectious diseases specialists in 7th day because no clinical response was achieved, and according to their advice, antibiotherapy was changed to ceftriaxone 2 × 1 gram daily, and then clinical signs and symptoms resolved. 

Imaging techniques-guided percutaneous drainage is preferable in cases of PLA. Surgical drainage is preferred in complex and multilocular abscess [21]. The patient in present case underwent cystectomy and cholecystectomy surgery because cyst was filling the right-upper quadrant of the abdomen and extended to umbilicus and contained a large number of cystic areas with different sizes.

In conclusion, infection of the hepatic hydatid cysts may occur and cause giant hepatic abscess as seen in our case. Furthermore, third-generation cephalosporins should be preferred instead of first- and second-generation cephalosporins in empirical treatment of abscess caused by *M. morganii* as it is naturally resistant to these antibiotics.
